# Clinical and economic effects of the transformation from an open to a laparoscopic center for colorectal surgery

**DOI:** 10.1007/s00423-024-03590-8

**Published:** 2025-01-15

**Authors:** Markus Zimmermann, Thaer S. A. Abdalla, Kai-Uwe Schlüter, Michael Thomaschewski, Tobias Keck, Erik Schlöricke

**Affiliations:** 1 Department of General Surgery, University Medical Center Schleswig Holstein, Campus Lübeck, Ratzeburger Alle 160, 23564 Lübeck, Germany; 2 Department of General Surgery, Westküstenklinikum Heide, Esmarchstraße 50, 25746 Heide, Germany

**Keywords:** Colorectal surgery, Laparoscopy, Open surgery, Colorectal carcinoma, Transition

## Abstract

**Purpose:**

The purpose of this study was to assess the feasibility of transitioning from open to laparoscopic surgery for colorectal carcinoma in a primary care hospital setting. Despite the recognized benefits of laparoscopic surgery in postoperative recovery and its demonstrated oncological equivalence, only a minority of patients (30–40%) in Germany undergo laparoscopic procedures, primarily due to concerns which, in addition to the perioperative quality data and economic aspects, focus on patient safety.

**Methods:**

Over a three-year period (2012–2014), the transformation process was observed in a colorectal cancer center. Data from 237 patients (115 laparoscopic; 122 open) were collected prospectively and analyzed retrospectively. Short-term outcomes, including demographic data, perioperative complications, and quality metrics, as well as long-term survival data, were included.

**Results:**

Laparoscopic surgery demonstrated several advantages. Postoperative intensive care needs decreased significantly (average length of stay: laparoscopic 1.2 days vs. open 2.5 days; *p* = 0.032). Hospital stays were also shorter following laparoscopic surgery (median laparoscopic 10 days vs. median open 14 days; *p* = 0.011). Quality of specimens, particularly lymph node retrieval, remained comparable (median laparoscopic = 18 vs. median open = 19). Survival data showed non- inferiority of the laparoscopic approach. Despite higher initial costs, laparoscopic surgery yielded cost savings of approximately 3150 € per case due to reduced intensive care and shorter hospital stays.

**Conclusion:**

In conclusion, this study demonstrates the feasibility of transitioning from open to laparoscopic oncologic colorectal surgery in a primary care hospital setting. The findings suggest that such a transition can be accomplished without compromising the quality of specimens, while also realizing cost savings and maintaining patient safety.

## Introduction

Since the late 1990s, minimally invasive surgery has been used in the surgical treatment of colorectal carcinoma [1]. The advantages in the perioperative course compared to the conventional open approach, such as the reduction of postoperative pain, the reduction of postoperative intestinal atony, earlier mobilization and consequently shorter hospital stays, are evident. The oncological equivalence of laparoscopy with open surgery, especially in the case of colon and rectal carcinoma, has already been proven by several trials [2–4]

Nevertheless, only about 30–40% of patients with colorectal carcinoma in Germany are treated laparoscopically [5]. The data is based on the recordings of health companies. The reasons for this are manifold. Still not all procedures for colorectal cancer are performed in colorectal cancer centers. On the other hand, oncological equivalence is still being discussed in some groups [4, 6]. Above all, however, the open procedure is the established procedure in many hospitals and a switch to laparoscopic care is not carried out for several reasons, which, in addition to the perioperative quality data and economic aspects, primarily focus on patient safety.

In this analysis, the transformation process over period of three years (2012 – 2014) from open to laparoscopic surgery is monitored in a center of primary care, during certification as a colorectal cancer center. Special attention is given to perioperative data such as the length of hospital stay, intensive care unit monitoring, complication data, quality of specimen as well as overall and disease-free survival.

## Methods

To access the transformation effects, the data of 237 patients who underwent surgical treatment with a colorectal cancer in the Westküstenklinikum Heide and Brunsbüttel gGmbH in the period from 01/2012 to 12/2014 were prospectively recorded and retrospectively evaluated. The data was collected as part of the certification process for the colon cancer center (DKG, OnkoZert). After completion of the certification in 2013, the data collection was continued. All patients included had consented to the collection and use of the collected data as part of the inpatient treatment contract. The tumor staging and the surgical procedure corresponded to the quality requirements of the professional societies and the current guidelines. The study was carried out with the approval of the responsible ethics committee (AZ 15-341A).

Applicable inclusion criteria were the presence of colorectal cancer and open or laparoscopic oncological curative resection of the tumor. The patient collective was divided into two groups. Group A included the laparoscopically operated patients and group B the patients who were treated conventionally via a midline laparotomy. The allocation to the groups was decided by the surgeon. In this process, patient characteristics, including age, body mass index (BMI), comorbidities, and general health status, were crucial in assessing suitability for different surgical methods. Another relevant consideration was the severity and location of the disease. Cases involving larger or more advanced tumors, high disease stage, or the presence of adhesions require open surgery to allow for thorough exploration and intervention. Finally, patient preference played a role in surgical planning but was not a frequent variable in the selection process.

In the further differentiation, a distinction was made between colon and rectal tumors to show differentiated course and quality parameters. In addition to demographic data, general and specific anamnestic parameters were recorded and evaluated. General medical history data included: Chronic obstructive pulmonary disease (COPD), myocardial infarction (MI), diabetes mellitus (DM), arterial hypertension (AHT), coronary artery disease (CHD), wound healing disorders in pre-op (WHD), hyperlipoproteinemia (HLP), prostate diseases (PD) and sleep apnea (SAP). The specific anamnestic data record included pre-existing underlying malignant diseases and previous abdominal operations. Furthermore, peri- and postoperative course data was recorded. The operating time (OT), the length of stay in hospital (LOS), the length of stay in the intensive care unit (ICU), the need for the administration of erythrocyte concentrates (ERY) and complications were recorded. Complications were classified into major and minor complications. Complications requiring surgical intervention were considered major complications. Major complications included anastomotic leaks (AI) and wound healing disorders (WHD). Minor complications included pneumonia (PM) and intestinal atonia (IA). In addition, the Clavien-Dindo score was recorded for all patients.

A special focus of this work was on the assessment and comparison of the quality of the surgical specimen. For this purpose, the number of lymph nodes, the distal resection margin and the circumferential resection margin were recorded and evaluated. The operations were performed by a total of three surgeons. Two of the surgeons had many years of experience in open colorectal oncological surgery. In addition, the surgeons were familiar with all common general laparoscopic procedures. The supervision of the implementation of laparoscopic oncologic procedures was monitored by a proven minimally invasive visceral surgeon, who had already performed more than 500 laparoscopic oncological colorectal resections before. The supervision was carried out with the assistance of the procedure with only temporary takeover of surgical steps. All cases were performed under supervision during the period of transformation.

The surgical standards of the procedures were performed according to the oncological guidelines at that time. This included central vessel ligation and adequate specimen length. Explicit CME procedures were not performed. Patients were positioned in supine position with both arms attached to the body using a vacuum mattress, regardless of the operation. Only sigmoid resection and deep anterior rectum resection were performed in lithotomy positioning. In addition, pneumatic calf compression cuffs were applied during each operation to minimize thrombotic or thromboembolic events. For right sided and central colon resections the specimen was recovered via a small supraumbilical median laparotomy. The anastomosis was usually created extracorporeally, side-to-side, isoperistaltic using a stapler. The auxiliary incision required for this is closed with a 4/0 serosubmucous suture. Only in the case of the left hemicolectomy is the anastomosis created end-to-end as a single-row hand suture and serosubmucous continuously with suture 4/0. The closure of the meso-slot was not performed.

For sigmoid and rectum resection the anastomosis was created using a trans anal circular stapler. To ensure a well-perfused anastomosis, the stapler anvil was only tied in if there was significant bleeding from the marginal arcade. The anastomosis was tested as an air–water test. In the case of anterior resections, a protective ileostomy is created in each case. If rectal extirpation was necessary, the specimen was retrieved transanally. A left-sided omental-plasty was created for both anterior resections and rectal extirpation.

### Statistics

In order to analyze the collected data, SPSS Version 26 from IBM (SPSS Inc., Chicago, IL, USA) was used. Continuous parameters were descriptively characterized by the following information: Median (Mdn), Minimum (Min), Maximum (Max) and Percentage (%). Dichotomous variables were examined for their frequency distribution and their percentage. The chi-square test (χ^2) was used to determine statistical relationships. With a sample size N < 20 and an expected cell count < 5, Fisher's exact test was used. To compare defined endpoints at the rational scale level between two groups, the Mann–Whitney U test (MWU) was performed for unpaired samples. The above tests were two-tailed with a probability of error of *p* = 0.05. A statistically significant relationship was assumed with a p-value ≤ 0.05. Survival analysis was performed using Kaplan–Meier estimations. Differences between groups were analyzed using the Log-Rank-Test.

## Results

**Fig. 1:**
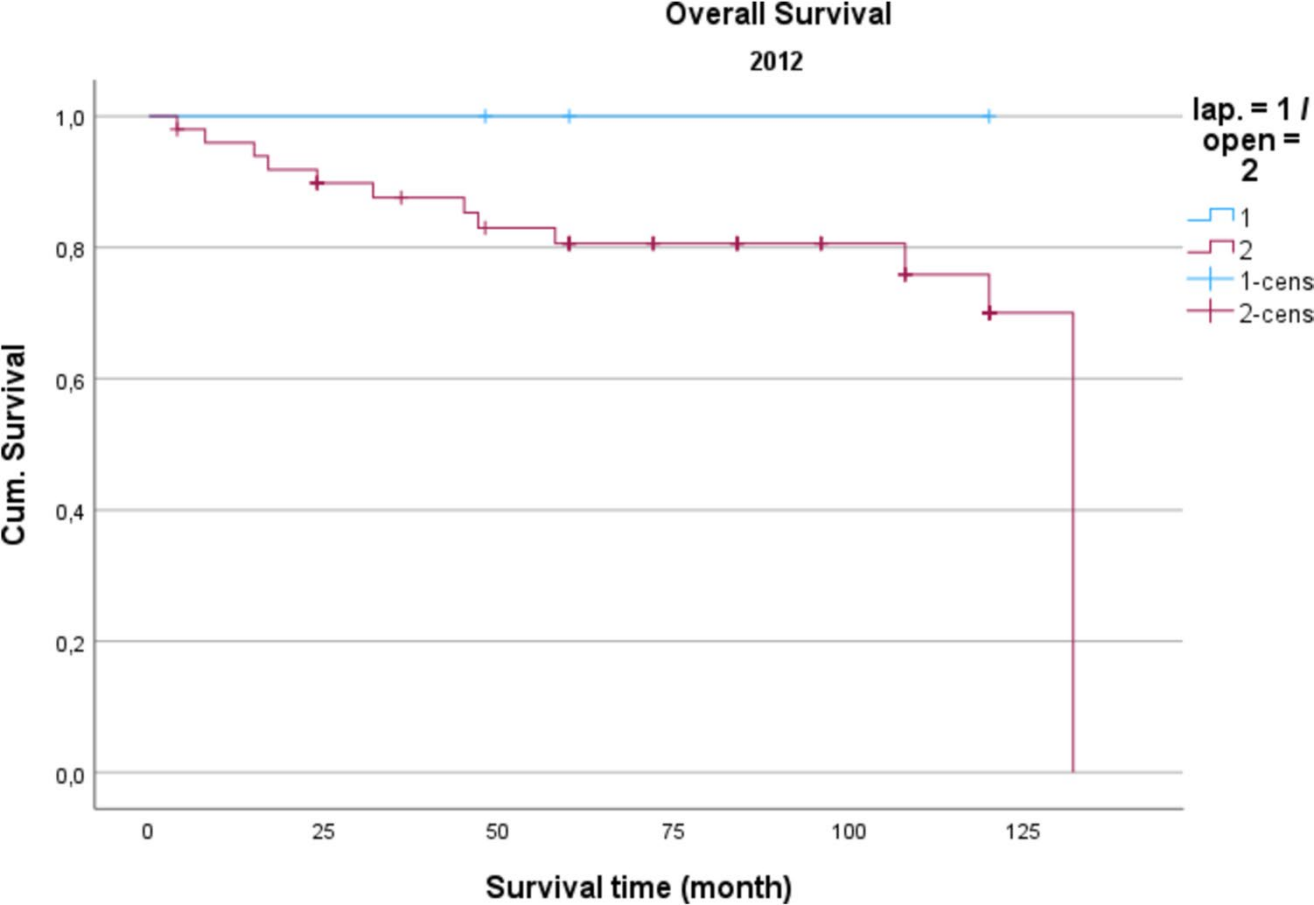
Overall survival 2012 *P* = 0.388

**Fig. 2:**
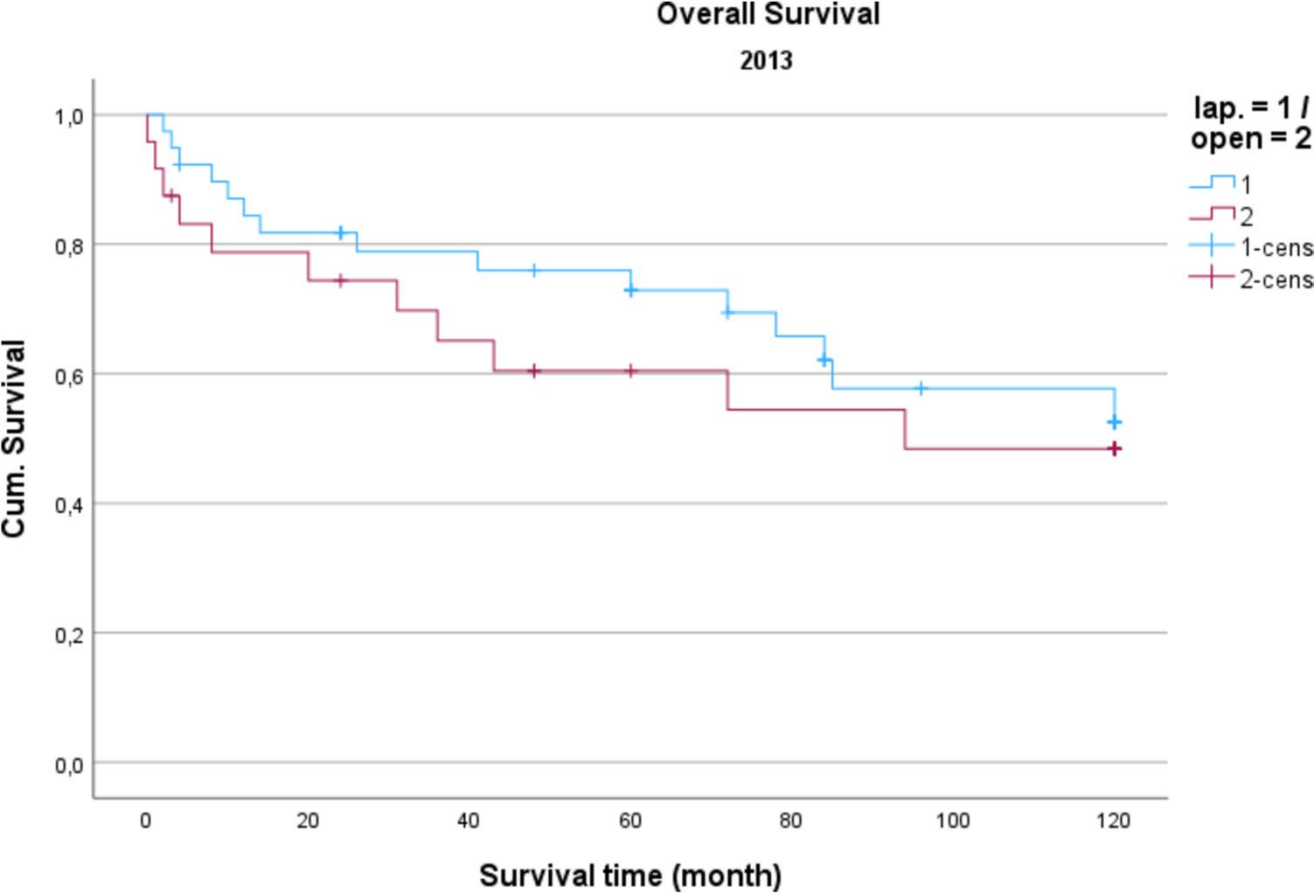
Overall survival 2013 *P* = 0.477

**Fig. 3:**
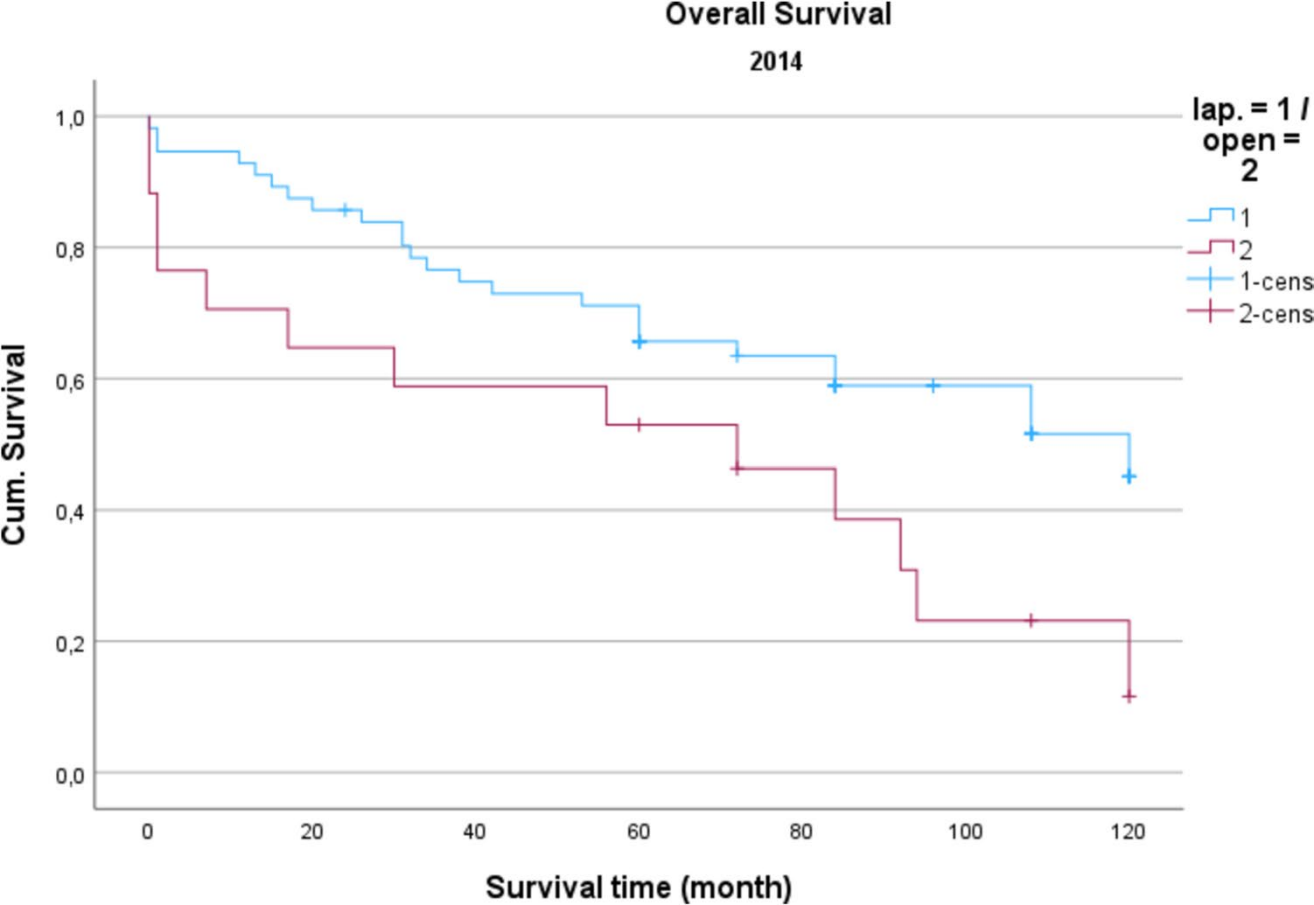
Overall survival 2014 *P* = 0.020

**Fig. 4:**
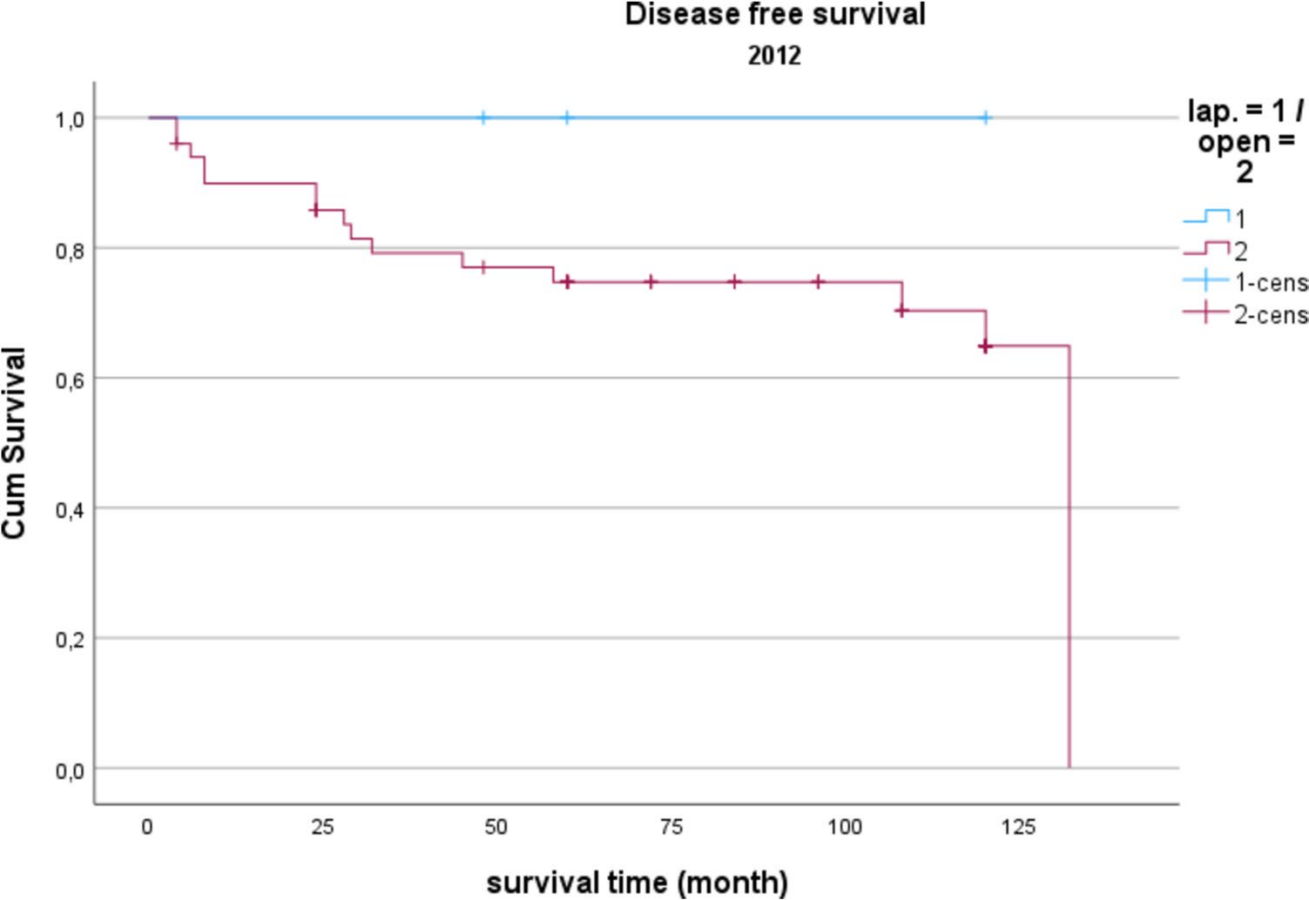
Disease free survival 2012 *P* = 0.388

**Fig. 5:**
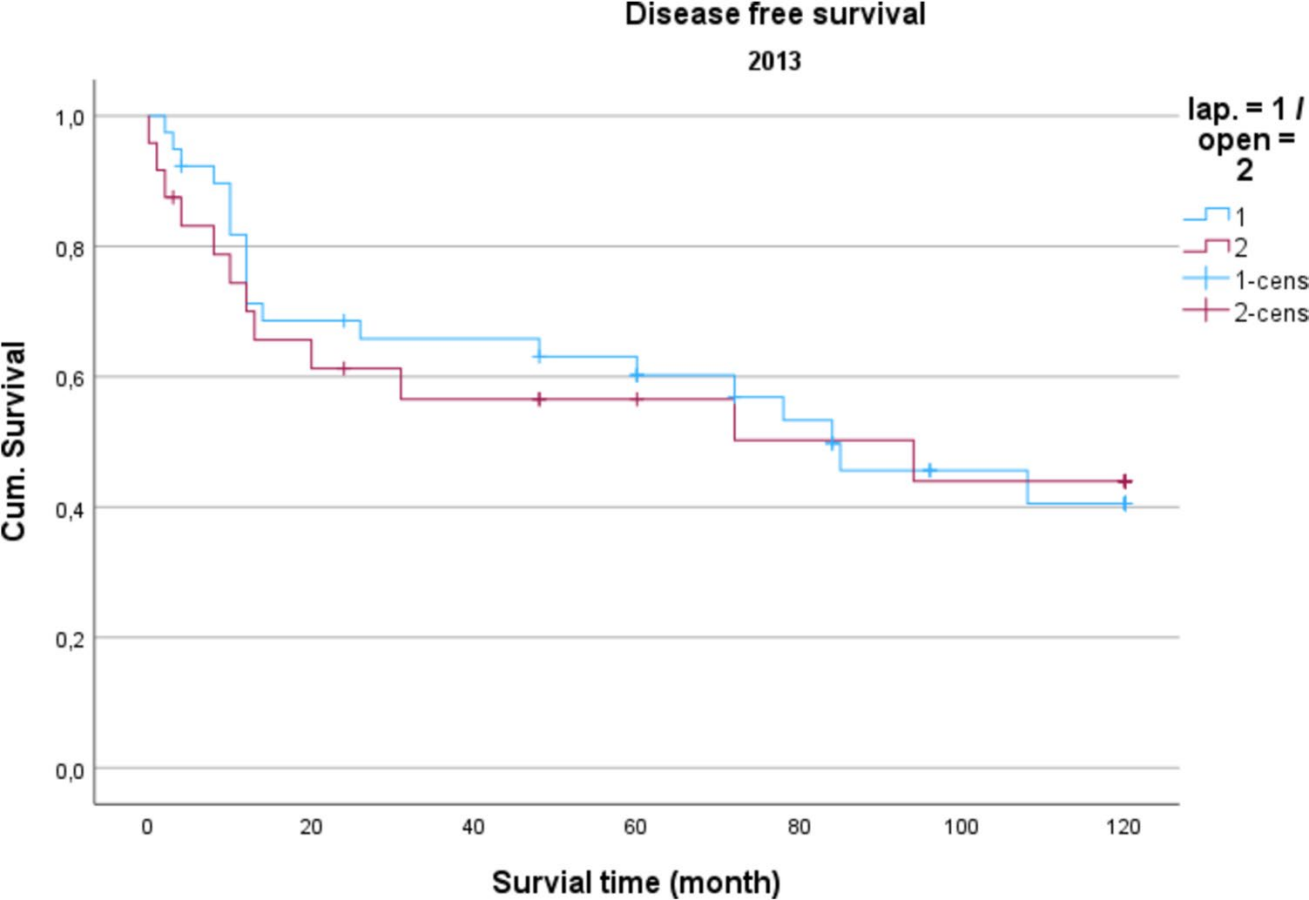
Disease free survival 2013 *P* = 0.388

**Fig. 6:**
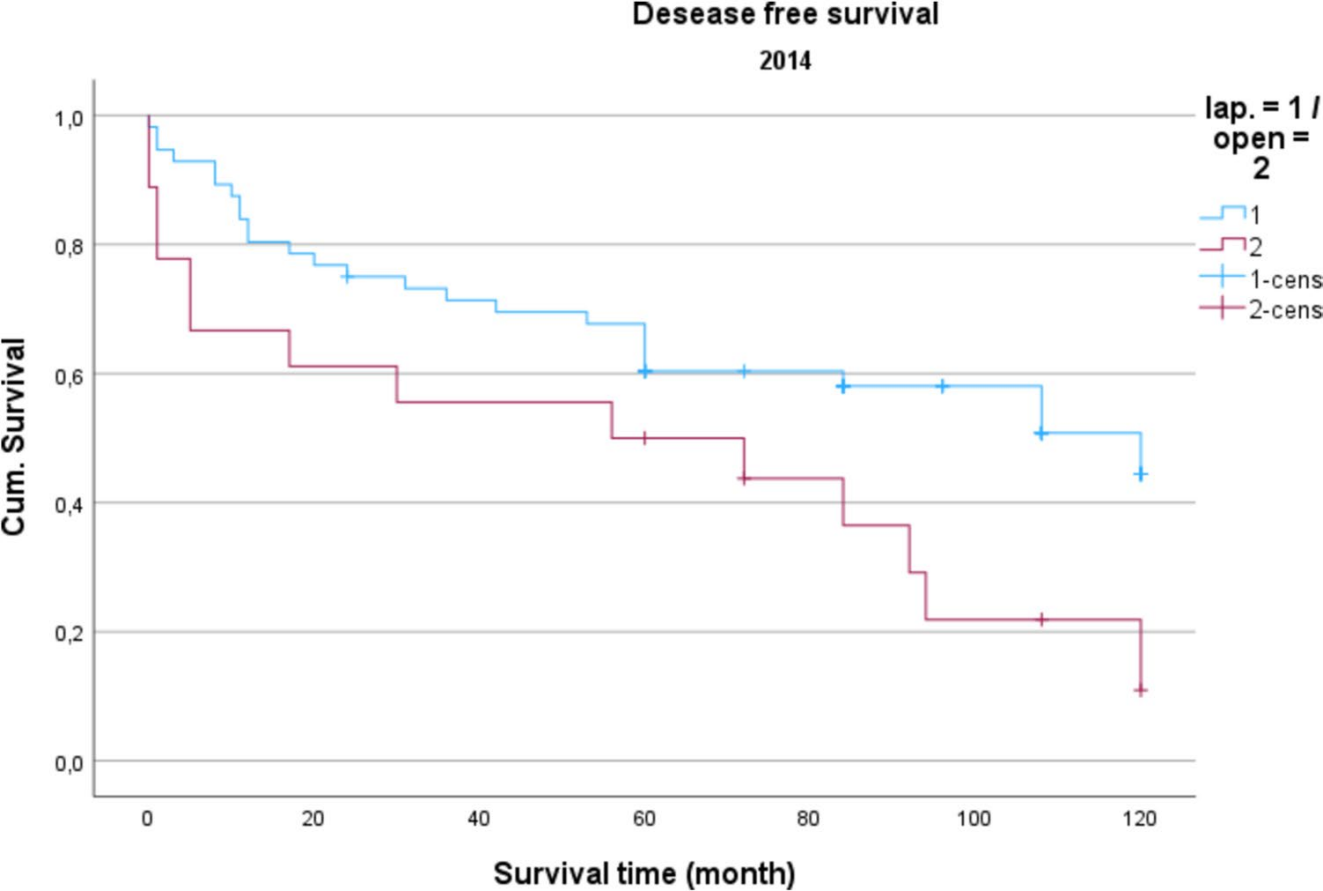
Disease free survival 2014 *P* = 0.020

Demographic data is analyzed in Table 1. The gender distribution showed a ratio of 100 women (42.2%) to 137 men (57.8%) in the total collective. The median age was 74.16 years, and the average BMI was 27 kg/m^2^. Concerning gender and BMI, there were no significant differences between open operated and laparoscopic operated patients. Regarding the age distribution of the patients in 2014, the conventional operated patients were significantly older than the laparoscopically operated patients (*p* = 0.039). Also, the American Society of Anaesthesiologists (ASA) Distribution showed no significant differences between the groups (Table 2). In the total collective, 17% of the patients had previously undergone an open abdominal operation and 4% had previous laparoscopic abdominal operation. In comparison, depending on the surgical procedure and the observation period, no significance for the existence of previous operations could be determined. Table 3 shows the distribution of tumor specifications in comparison between patients treated laparoscopically and openly referring to the observation period. In the overall collective and over the entire observation period, most patients had a postop. UICC stage III (mean = 25.6 cases per year; 33.2% of all cases). When comparing the open vs. laparoscopic groups referring to the observation period, no significance could be demonstrated when comparing the distribution pattern according to the tumor stage. Analyzing the perioperative parameters, Table 4 shows the median operation time according to the observation periods. Likewise, in the previous parameters, no significant differences between the two techniques could be found. Further postoperative parameters included in the analysis of the length of hospital stay (LOS), the duration of the need for epidural anesthesia (epidural catheter, PDA) (Table 5) and the complications according to the Clavien-Dindo-Score (Table 6). For the required length of stay in hospital, a significantly shorter stay was shown for laparoscopically operated patients in 2013 compared to the open operated group (*p* = 0.011). In the years 2012 and 2014 no significant difference could be determined but a shorter LOS for the laparoscopic group in 2014 was obvious. The analysis of the postoperative stay on the intensive care unit (ICU) showed a significant shorter time for the laparoscopic group in the years 2013 and 2014. No significance could be observed between the two procedures the length of a PDA. The median in 2012 was 2 days for laparoscopic surgery and 1 day for open surgery. In 2013, the median for both procedures was 2 d. In 2014, 2.5 days were required for laparoscopic surgery vs. 3 days for open procedures. A PDD was applied in 85 patients (72.0%) in the laparoscopic group and in 69 patients (59.8%) in the open group. Complications from open and laparoscopic surgery were evaluated using the Clavien-Dindo-Score (Table 6). This analysis revealed no significant advantage for either method. A total of 163 patients (68.7%) could be discharged with a score of 0 (no complication). A total of 74 patients (31.2%) had a Clavien-Dindo score of 1–3. Of these 74 patients, 34 patients underwent laparoscopic surgery, and 40 patients underwent open surgery. The scores 4 and 5 could not be observed over the observation period. The complications were also differentiated to minor and major complications. Major complications were defined as the need for surgical or endoscopic re-intervention and listed in Table 7.. Over the entire observation period and independent of the surgical procedure, 12 patients (7.6%) with colon carcinoma developed an anastomotic leak and 8 patients (5.4%) experienced a complication of impaired wound healing. In patients with rectal carcinoma, the rate of anastomotic leaks was 12.3% (n = 10) and of wound healing disorders 5.4% (n = 4). No significant difference could be shown when comparing the surgical procedures regarding major complications. In 2012, an anastomotic leak was observed in 10.0% (n = 2) rectal cancer patients who underwent open surgery. In 2012, no anastomotic leak was observed in laparoscopically operated rectal cancer patients. The open operated colon carcinoma patients in 2012 were affected by an anastomotic leakage in 6.0% of the cases. In the laparoscopic group, no anastomotic leak was observed in the patients with colon carcinoma in 2012 either. In 2013, the rate of anastomotic leaks in patients with rectal cancer was 21.1% (n = 4) in the laparoscopic group and 11.1% (n = 1) in the open group. Wound healing disorders could be observed in one case this year after open rectal carcinoma resection. In 2014, patients after rectal carcinoma resection were affected by an anastomotic leak in 7.4% (n = 2) of the cases after laparoscopic surgery and in 25.0% (n = 1) of the cases with open surgery. In the same observation year, after resection of a colon carcinoma, 2 anastomotic leaks (11.8%) were recorded in the open operated group and 4 (10.3%) in the laparoscopic operated group. To monitor the surgical quality the number of lymph nodes and the safety margin of the specimens were included in the analysis. The distal safety margin was evaluated for colon carcinomas and the distal and circumferential safety margins for rectal carcinomas. Also, the MERCURY grade and the R- status of rectal resections were analyzed. (Table 8, 9). Concerning all analyzed quality parameters for rectal and colonic resections, no statistically significant differences between both surgical techniques could be demonstrated. To evaluate the economic dimension of both procedures a detailed cost calculation was performed (Table 10). This results in additional costs for laparoscopic operations per case due to the additionally required material of 122 euros for colon resection (0.84% ​​of the total costs/case) and 137 euros for rectal resection (0.94% of the total costs/case) with the same revenues according to the DRG regardless of the surgical procedure. These additional costs can be compensated for by shortening the LOS and the ICU stay. In this analysis, the median LOS in the last year of transformation was 11 days for laparoscopically operated patients, while the open operated patients required a median LOS of 14 days. With a median reduction in LOS of 3 days and hospital costs for a peripheral bed of around 360 euros per bed/d (data from 2021, Department of Controlling, WKK Heide), this results in savings of 1,080 euros/case. In addition, over the entire observation period after laparoscopic surgery compared to open surgery, treatment duration in the intensive care unit was reduced by a median of 1.3 days. With costs for an intensive care unit of 1,696 euros/day (data from 2021, department for controlling, WKK Heide), this results in additional savings of 2,205 euros/case, which leads to an effective saving of 3,147 euros/case. Finally, Figs. 1, 2 and 3 show the Overall survival (OS) and Figs. 4, 5 and 6 the Disease-free survival (DFS) of the laparoscopic and open group stratified by the operation year 2012 to 2014. The log rank test comparing the Kaplan–Meier-Curves showed no significant differences for the years 2012 and 2013 whereas the curves for the year 2014 show a significant longer OS and DFS for the laparoscopic group.

**Table 1:** Demographic data of laparoscopic and open resected patients referring to the observation period

	2012			2013			2014		
	lap	open	*p*-value	lap	open	*p*-value	lap	open	*p*-value
Gender									
male	5 (28.6 %)	37 (52.9 %)	0.347	22 (52.4 %)	16 (51.6 %)	0.948	44 (66.7 %)	13 (61.9 %)	0.689
female	2 (71.4%) 7	33 (47.1%)		20 (47.6%)	15 (48.4%)		(33.3 %)	8 (38.1%)	
Sum	7	70		42	31		66	21	
age	77 (62–85)	73 (30–91)	0.689	72 (46–87)	74 (42–92)	0.763	73 (39–87)	76 (39–83)	0.039
BMI, kg/m2	27 (25–32)	28 (19–48)	0.970	27 (21–40)	26 (19–36)	0.863	27 (17–35)	27 (19–34)	0.356

**Table 2:** ASA Classification of laparoscopic and open resected patients referring to the observation period

	2012			2013			2014		
	lap	open	total	lap	open	total	lap	open	total
ASA I	0 0,0%	2 2,9%	2 2,6%	0 0,0%	0 0,0%	0 0,0%	0 0,0%	0 0,0%	0 0,0%
ASA II	1 14,3%	18 25,7%	19 24,7%	16 38,1%	9 29,0%	25 34,2%	17 25,8%	5 23,8%	22 25,3%
ASA III	6 85,7%	49 70%	55 71,4%	26 61,9%	18 58,1%	44 60,3%	48 72,7%	14 66,7%	62 71,3%
ASA IV	0 0,0%	1 1,4%	1 1,3%	0 0,0%	4 12,9%	4 5,5%	1 1,5%	1 4,8%	2 2,3%
total	7	70	77	42	31	73	66	21	87
p-value	0,836			0,053			0,246

**Table 3:** Tumor specifics of laparoscopic and open resected patients referring to the observation period4

Jahr	2012			2013			2014		
UICC	lap	offen	ges	lap	offen	ges	lap	offen	ges
I	3 (42,9 %)	13 (18,8 %)	16 (21,1 %)	7 (16,7 %)	5 (16,1 %)	12 (16,4 %)	16 (25,0 %)	2 (9,5 %)	18 (21,2 %)
II	0 (0,0 %)	18 (26,1 %)	18 (23,7 %)	8 (19,0 %)	10 (32,3 %)	18 (24,7 %)	22 (34,4 %)	(42,9 %)	31 (36,5 %)
III	0 (0,0 %)	19 (27,5 %)	19 (25,0 %)	24 (57,1 %)	9 (29,0 %)	33 (45,2 %)	18 (28,1 %)	7 (33,3 %)	25 (29,4 %)
IV	4 (57,1 %)	19 (27,5 %)	23 (30,3 %)	3 (7,1 %)	7 (22,6 %)	10 (13,7 %)	8 (12,5 %)	3 (14,3 %)	11 (12,9 %)
p-value	0,062			0,058			0,515

**Table 4:** Operation-time referring to the observation period (median)

Operation time (min)	2012	2013	2014
Colon laparoscopic	128	186	183
Colon open	201	192	183
p-value	0,398	0,460	0,776
Rectum laparoscopic	203	183	179
Rectum open	206	200	180
p-value	0,286	0,308	0,214

**Table 5:** Postoperative parameters referring to the observation period

	2012		2013		2014		
	lap	offen	lap	offen	lap		offen
Length of stay (d) -Median -Minimum -Maximum	16,0 6,0 30,0	14,0 5,0 46,0	10,0 4,0 25,0	14,0 8,0 136,0	11,0 7,0 36,0		14,0 7,0 28,0
p-value	1,000		0,011		0,091		
Lenght of ICU-stay (d) -Median -Minimum -Maximum	1,0 0 2,0	2 0 14	0 0 5	3 0 31	0 0 16	2 0 14	
p-value	0,394		< 0,001		0,001		
Lenght of PDA (d) -Median -Minimum -Maximum	2,0 0 2,0	1,0 0 4,0	2,0 0 4,0	2,0 0 5,0	2,5 0 5,0		3,0 0 4,0
p-value	0,593		0,057		0,078

**Table 6:** Clavien-Dindo-score of laparoscopic and open resected patients referring to the observation period

Clavien-Dindo	2012			2013			2014		
	lap	open	total	lap	open	total	lap	open	total
0	5 71,4%	47 67,1%	52 67,5%	30 71,4%	23 74,2%	53 72,6%	46 69,7%	12 57,1%	58 66,7%
1	0 0,0%	8 11,4%	8 10,4%	7 16,7%	2 6,5%	9 12,3%	7 10,6%	2 9,5%	9 10,3%
2	2 28,6%	9 12,9%	11 14,3%	4 9,5%	3 9,7%	7 9,6%	8 12,1%	5 23,8%	13 14,9%
3a / 3b	0 0,0%	6 8,6%	6 7,8%	1 2,4%	3 9,7%	4 5,5%	5 7,6%	2 9,5%	7 8,0%
p-value	0,472			0,353			0,588

**Table 7:** Major complications of laparoscopic and open resected patients referring to the observation period

Major Complications	2012			2013			2014		
	lap	offen	ges	lap	offen	ges	lap	offen	ges
Anastomotic Leak Colon									
-N	0	3	3	0	3	3	4	2	6
-%	0,0 %	6,0 %	5,5 %	0,0 %	13,6 %	6,7 %	10,3 %	11,8 %	10,7 %
* p*-value	0,573			0,067			0,887		
Woundhealing disturbance Colon									
-N	1	2	3	2	2	4	1	0	1
-%	20,0 %	4,0 %	5,5 %	8,7 %	9,1 %	8,9 %	2,6 %	0,0 %	1,8 %
* p*-value	0,133			0,963			0,505		
Anastomotic Leak Rectum									
-N	0	2	2	4	1	5	2	1	3
-%	0,0 %	10,0 %	9,5 %	21,1 %	11,1 %	17,9 %	7,4 %	25,0 %	9,7 %
* p*-value	0,740			0,521			0,267		
Woundhealing dis. Rectum									
-N	0	2	2	0	1	1	1	0	1
-%	0,0 %	10,0 %	9,5 %	0,0 %	11,1 %	3,6 %	3,7 %	0,0 %	3,2 %
* p*-value	0,740			0,139			0,696		
Revision Colon									
-N	2	3	3	1	3	4	4	2	6
-%	40,0 %	6,0 %	9,1 %	4,3 %	13,6 %	8,9 %	10,3 %	11,8 %	10,7 %
* p*-value	0,012			0,274			0,867		
Revision Rectum									
-N	0	3	3	1	1	2	0	2	2
-%	0,0 %	15,0 %	14,3 %	5,3 %	11,1 %	7,1 %	0,0 %	50,0 %	6,5 %
* p*-value	0,676			0,575			0,001

**Table 8:** Quality parameters rectum of laparoscopic and open resected patients referring to the observation period

QUALITY PARAMETERS RECTUM	2012		2013		2014	
	lap	offen	lap	offen	lap	offen
Lymphnodes total -Medi -Min -Max	19 19 19	18 0 29	17 12 37	20 11 34	16 6 56	26 13 43
p-value	0,571		0,357		0,245	
Distal resection margin (cm) -Med -Min -Max	4 4 4	2 5 6	1 0 4,5	3 8 8	2,5 0,5 11	3 1 7
p-value	0,381		0,076		0,647	
Circumferential resection margin (cm) -Med -Min -Max	2 2 2	1,5 0 2,5	1,5 0 2,5	1 0 3	1,5 0 2,5	1,5 1,5 1,5
p-value	0,526		0,152		0,906	
R-status 0 1	50,0 50,0	94,7 5,3	94,7 5,3	88,9 11,1	96,3 3,7	92,8 7,2
p-value	0,186		0,818		0,511	
M.E.R.C.U.R.Y. -grade 1 2 3	50,0 50,0 0	89,1 4,3 6,6	84,2 10,5 5,3	77,8 11,1 11,1	81,5 11,1 7,4	75 25 0
p-value	0,186		0,876		0,659

**Table 9:** Quality parameters colon of laparoscopic and open resected patients referring to the observation period

QUALITY PARAMETERS COLON	2012		2013		2014	
	lap	offen	lap	offen	lap	offen
Lymphnodes total -Med -Min -Max	13 8 28	19 8 60	22 9 62	18 8 51	22 7 80	18 3 54
p-value	0,089		0,267		0,307	
Distal resection margin (cm) -Med -Min -Max	6,5 1,5 11	11 1,5 28	9,5 2,5 20	7 0 25	9,5 2 25	10 2 28
p-value	0,295		0,257		0,973

**Table 10:** Costcalcualtion of material costs for colonic and rectal resections

Material costs colonic resection general	Material costs rectal resection general
−1 × Harmonic 36 cm 374 Euro	−1 × Harmonic 36 cm 374 Euro
−1 × EndoGia Magazine 45 mm 179 Euro	−1 × EndoGia Magazine 45 mm 179 Euro
−1 × Power Shell 125 Euro	−1 × Power Shell 125 Euro
Total 678 Euro	−1 × Circular Stapler 471 Euro
	Total 1149 Euro
Extra costs colonic resection laparoscopy	Extra costs rectal resection laparoscopy
−1 × Lap suction 18 Euro	−1 × Lap suction 18 Euro
−1 × Alexis retractor 50 Euro	−1 × Alexis retractor 50 Euro
−1 × Gas insufflation set 4 Euro	−1 × Gas insufflation set 4 Euro
−1 × 5 mm Trocar (Duo) 20 Euro	−1 × 5 mm Trocar (Duo) 20 Euro
−2 × 11 mm Trocar 30 Euro	−2 × 11 mm Trocar 30 Euro
Total 122 Euro	−1 × 12 mm Trocar 15 Euro
	Total 137 Euro

## Discussion

The transition from open surgery to the adoption of laparoscopic techniques in the treatment of colorectal cancer has manifested as a transformative journey, linked with both clinical and economic dimensions. This study contributes substantially to the ongoing discourse by offering an analysis of the implications associated with this paradigm shift, enhancing the understanding of the multifaceted landscape in colorectal surgery. The recurrent debate about the adoption of laparoscopic surgery in colorectal carcinoma treatment has persisted despite the evident advantages documented since the late 1990s [7].This study, conducted over a three-year period (2012–2014) at a colorectal cancer-certified primary care center, provides a comprehensive analysis of the transformation process. The primary motivation behind the study initiates from the observation that, despite established benefits such as reduced postoperative pain, shorter hospital stays, and earlier mobilization, only 30–40% of colorectal cancer patients in Germany undergo laparoscopic procedures [5]. This discrepancy is attributed to concerns about oncological equivalence, and concerns about patient safety. The data, collected prospectively and analyzed retrospectively, encompasses 237 patients undergoing colorectal surgery. Notably, the operations were executed by three surgeons, each bringing a distinct skill set — one proficient in minimally invasive surgery and two with extensive experience in open oncological surgery. This degree of expertise underscores the pragmatic approach adopted during the transition period. Key results showed a significant reduction in the need for postoperative intensive care, symbolizing enhanced recovery following laparoscopic procedures. The reduction in the average length of stay from 2.5 days to 1.2 days further substantiates the enhanced postoperative recovery associated with laparoscopic colorectal surgery. Notably, the length of hospital stay following laparoscopic surgery emerges as significantly shorter than the open approach, correlating with existing literature that underscores the efficiency of minimally invasive approaches in colorectal surgery [8–10]. Quality metrics, a cornerstone in surgical assessments, emerge unscathed during the transformation. Therefore, the study examines the number of lymph nodes in surgical specimens, a crucial parameter in oncological outcomes. The consistent high quality of specimens, irrespective of the surgical approach or the observation period, shows the standards maintained throughout the transition. This effect also is seen in literature even in advanced rectal cancer [11]. Moreover, quality parameters such as the Distal and Circumferential Resection Margin do not differ between groups. Also, the R-Status and the MERCURY grade showed no significant differences between the laparoscopic and the open approach ovar all three years. Furthermore, the study focuses on the stability of major complications, with anastomotic leaks exhibiting no significant differences between laparoscopy and open surgery. This commitment to surgical quality stands for a successful transformative process and is a cornerstone for laparoscopic adoption. Even for patients aged 80 and older Chung et al. could demonstrate similar effects showing advantages for the laparoscopic approach and making it the approach of choice [12].

Regarding long-term survival, only the year 2014 shows differences in the OS as well as the DFS. Curves show a significant advantage of the laparoscopic approach. This could be a result of an selection bias, but could also result from the advantages of the laparoscopic technique, which in literature also showed relevant survival benefits resulting from modulated immune response [13] as well as the tumor cell spread [14]. In summary, the laparoscopic approach shows no inferiority in the underlying data, which is in line with the well-known randomized prospective studies addressing this issue [7, 15].

Economic considerations form another important part of the study, addressing concerns related to the financial implications of embracing laparoscopic techniques. Despite the upfront investment costs and higher material expenses associated with laparoscopy, the study introduces a compelling narrative of economic viability. The reduction in intensive care monitoring time and hospital stay not only offsets the initial costs but results in a net economic advantage. The projected savings of around 3150 euros per case for laparoscopic surgery underscore the fiscal prudence inherent in this paradigm shift. These results are in line with comparable international studies also suggesting the cost effectiveness of the laparoscopic approach [16–18]. A cost-effectiveness analysis made in the USA for patients older than 65 years also revealed a cost advantage of $3276 per case and therefore resulting in a comparable saving amount [19].

However, a nuanced interpretation necessitates acknowledgment of certain limitations inherent in the study. The focus on a single center introduces a potential limitation in terms of generalizability. Surgeon experience, albeit briefly mentioned, could potentially influence outcomes, warranting a more in-depth exploration of its impact. Additionally, the three-year observation period, while providing valuable short- and long-term insights, prompts contemplation about the sustainability of the observed benefits over a more extended timeframe. Moreover, the limited number of patients, the retrospective study design and the group allocation by surgeon preference limits the validity of the results.

In conclusion, this study offers an analysis of the transformative process from open to laparoscopic colorectal surgery, including both clinical and economic dimensions. The consolidation of positive clinical outcomes, economic advantages, and the constant surgical quality emerges as a powerful narrative favoring for the continued adoption of laparoscopic techniques. As the healthcare landscape navigates the difficulties of evidence-based practices and endeavors for cost-effective healthcare delivery, the study positions laparoscopic colorectal surgery as not just a pragmatic but a strategic choice. The sustained success of this transformative paradigm shifts relies on ongoing training, strict adherence to established guidelines [20], and the implementation of robust quality monitoring mechanisms, ensuring that the benefits outlined in this study show the feasibility of a save transition process.

## Data Availability

No datasets were generated or analysed during the current study.
